# Development of diabetes complications within coordinated and structured primary health care: a 10-year retrospective cohort study in Germany

**DOI:** 10.3399/BJGPO.2024.0061

**Published:** 2024-08-07

**Authors:** Kateryna Karimova, Catriona Mairi Friedmacher, Dorothea Lemke, Anastasiya Glushan

**Affiliations:** 1 Institute of General Practice, Goethe University Frankfurt, Frankfurt, Germany

**Keywords:** structured and coordinated primary health care, diabetes mellitus, diabetes complications, chronic disease

## Abstract

**Background:**

Diabetes mellitus is a growing, costly, and potentially preventable public health issue. In 2004, Germany introduced the GP-centred healthcare programme to strengthen primary care.

**Aim:**

To assess the hazards of the most common diabetes-related complications in patients enrolled in GP-centred health care in comparison with usual primary care.

**Design & setting:**

A retrospective cohort study based on German claims data (4 million members) from 2011–2020.

**Method:**

In total, 217 964 patients with diabetes were monitored from 2011–2020. Endpoints were blindness, amputation, myocardial infarction, stroke, coronary heart disease, dialysis, hypoglycaemia, and all-cause mortality. Cox proportional-hazards regression models were used for multivariable analysis and adjusted for sociodemographic, practice, and disease-specific characteristics.

**Results:**

Compared with usual care (*n* = 98 609 patients), GP-centred health care (*n* = 119 355 patients) showed a relative risk reduction of blindness of 12%, and amputation of 20% over 10 years. The estimated impact of GP-centred health care on myocardial infarction, stroke, coronary artery disease, dialysis, and all-cause mortality is significantly favourable in comparison with usual care. However, the proportional risk of hypoglycaemia (+1.2%) in the interventional group is higher than in usual care.

**Conclusion:**

Enrolment in GP-centred health care appears to result in a consistent reduction of the relative risk of diabetes-related complications over 10 years. The significant difference in contrast to usual care may be explained by robust, structured primary care provision, including the diabetes disease management programme, and improved coordination and networking of care within primary and secondary care.

## How this fits in

Structured and integrated primary health care plays a crucial role in the effective management of diabetes mellitus. The GP-centred healthcare programme (Hausarztzentrierte Versorgung; HZV), aims to enhance health care for patients with chronic diseases (especially diabetes), improve patient-oriented outcomes, and delay the development of diabetes-related complications. This study demonstrates a consistent reduced risk of the development of diabetes-related complications over a 10-year observation period. Policies, such as the GP-centred healthcare programme, could be introduced into other countries with a functioning primary care system but fragmentary or lacking chronic disease management programmes.

## Introduction

Diabetes mellitus is a major cause of morbidity and mortality worldwide.^1^ Diabetes is associated with microvascular and macrovascular complications, in particular coronary artery disease, stroke, renal failure, blindness, and amputation.^2,3^ In 2021, it was estimated that, globally, 537 million people were living with diabetes, and that this number was projected to reach 643 million by 2030, and 783 million by 2045.^4,5^ Global health expenditure on diabetes in 2021 was already approaching 1 trillion USD.^5^

The main aims of diabetes care are to reduce the risk of short- and long-term complications, increase longevity, and improve health-related quality of life.^5^ The majority of international guidelines relating to the medical care of patients with diabetes recommend a structured and coordinated primary care approach to diabetes management, which emphasises patient-centred multidisciplinary care, integrated long-term treatment approaches to diabetes and comorbidities, and ongoing collaborative communication and goal setting between all team members with an effective system of referral and feedback.^2,6,7^ GPs play a central role worldwide in administering and coordinating the provision of care for their patients with diabetes.^8,9^

Most GPs in Germany work in small independent practices (one or two physicians) with no involvement in large managed healthcare plans or collaborative healthcare systems. Two attempts to integrate chronic care models into this system were the nationwide disease management programmes (DMPs)^10^ and GP-centred healthcare programmes (established in several federal states). The German DMPs have been designed to improve the quality of care for patients with chronic diseases (diabetes mellitus, asthma, chronic obstructive pulmonary disease [COPD], and coronary artery disease), to reduce complications, to improve patient-oriented outcomes, and to improve cost-efficiency.

The GP-centred healthcare programme, 'Hausarztzentrierte Versorgung’ (HZV), aims to enhance health care for patients with chronic conditions (not only those included in DMPs) and complex healthcare needs (for example, those requiring long-term care). Doctors who have registered to participate in GP-centred health care are required to comply with the following measures with respect to diabetes care: participation in structured quality networks on drug therapy ‘quality circles’ (that is, small groups of physicians who receive feedback on their prescribing, evidence-based information, and plan quality improvement measures); rigorous application of evidence-based guidelines developed for use in primary health care; and obligatory participation in all DMPs. Patients enrol voluntarily in HZV with a personal GP, who coordinates referrals (gate-keeping). Patients who are enrolled in the HZV programme can be, supplementary to this, enrolled in ambulatory specialist health care (cardiology, diabetology, orthopaedic, psychological care, and so on) thus receiving intensive specialist health care in close collaboration with GPs (coordinated role), with an obligatory system of referral and feedback, and close communication between GPs and specialists. The GPs participating in the HZV programme are better remunerated and have an increased budget.

The aim of this retrospective cohort study was to evaluate the development of diabetes-related complications over 10 years, comparing patients enrolled in the GP-centred healthcare programme with patients receiving usual GP care. Earlier results for the same study question, relating to the observation period from 2011–2014, have been previously published.^2^

## Method

### Setting and study design

A retrospective closed cohort study was carried out as part of an evaluation of HZV in the German regional state of Baden-Württemberg and was fully approved by the ethics committee of Frankfurt University Hospital. All participants in the study were insured by Allgemeine Ortskrankenkasse Baden-Württemberg (AOK-BaWü).

### Data extraction, endpoints, and covariates

Administrative data (insurance claims data) were provided by AOK-BaWü for the years 2010–2020. AOK BaWü is the largest statutory healthcare fund of Southwest Germany including 4 million members. The claims data of AOK BaWü were collected from:

1. hospital inpatient data from 2010–2020 of insured members of AOK BaWü;
2. (outpatient) ambulatory care data (GPs and specialists) from 2010–2020 of insured members of AOK BaWü;
3. prescription data from 2010–2020 of insured members of AOK BaWü; and
4. demographic data on insured members, which includes age and sex of patients.

The German claims data do not include laboratory data, such as HbA1c, blood pressure, body mass index (BMI), and serum creatinine. International Classification of Diseases (ICD)-coded clinical diagnoses data were used to collect data on diabetes complications as incident (newly occurred) primary endpoints. The primary endpoints of this analysis are shown in Table 1.

**Table 1.** Primary endpoints

Primary endpoints	ICD-10 code^a^
Dialysis	Z49.0; Z49.1; Z49.2; Z99.2; N18.5
Blindness	H54.0; H54.4
Amputation	Z89.4; Z89.5; Z89.6; Z89.7
Myocardial infarction	I21
Stroke	I64; I61; I63
Cardiovascular disease	I21; I22; I23; I24; I25
Hypoglycaemia	E78
All-cause mortality	Reported by the AOK monthly

^a^Reported outpatient or inpatient primary diagnosis codes. AOK = Allgemeine Ortskrankenkasse. ICD-10 = International Classification of Diseases, Tenth Revision.

### Participants

To be included in the study, participants had to have type 1 or type 2 diabetes mellitus (ICD-10 codes E10–E14), be ongoing AOK-BaWü members, live in Baden-Württemberg, be aged ≥18 years, not be participating in other healthcare programmes, and satisfy further administrative inclusion criteria. Patients in the HZV group had to enrol in the programme before 1 January 2011; patients in the usual care group had to have an identifiable GP. Patients who switched to other healthcare funds at the baseline were excluded. We considered deceased patients to be censored observations until time of death. We had no missing values because of our inclusion criteria (available insurance data) and study design.

### Statistical analysis

The period before the occurrence of a clinical endpoint was calculated in days (beginning on 1 January 2011) until the occurrence of an index event (primary endpoint). A Cox proportional-hazards regression model was used to analyse the time until an endpoint was reached (Table 1) using the covariates listed in Table 2. The care level, general morbidity score, using the Charlson Comorbidity Index (CCI), and special diabetes morbidity score, using the adapted Diabetes Complications Severity Index (aDCSI), were annually computed and used as time-dependent variables in the Cox model.

**Table 2.** Covariates of the analysis (ascertained at baseline)

Covariates	Description
Age	In decades
Sex	Categorical or reference male
DM DMP participation	Binary, 0/1
Care level^a,b,c^	Binary, 0/1
Comorbidities	Arterial hypertension and ischaemic heart disease (binary, 0/1)
Charlson Comorbidity Index^a,b^	General comorbidity score: 0–18
adapted Diabetes Complications Severity Index^a,b^	Index ranges from 0–13, consisting of scores (0, 1, or 2) for seven diabetes-related complications^d^
Practice variables	Practice size, joint practice (binary, 0/1), and urban or rural (binary, 0/1)

^a^These covariates were computed and included in the statistical analysis on an annual basis. ^b^Time-dependent variables. ^c^Formal level of care designation based on the degree of impairment to the independence or abilities of the person needing care. ^d^Categories: retinopathy, nephropathy, neuropathy, cerebrovascular disease, cardiovascular disease, peripheral vascular disease, and metabolic syndrome. DM = diabetes mellitus. DMP = disease management programme.

Hazard ratios (HRs) are presented as point estimates with 95% confidence intervals (CIs) and *P*-values (*P*<0.0001). As this was an exploratory study, no adjustment for multiple testing was performed. All analyses were implemented using SAS (version 9.4).

## Results

**Figure 1.**
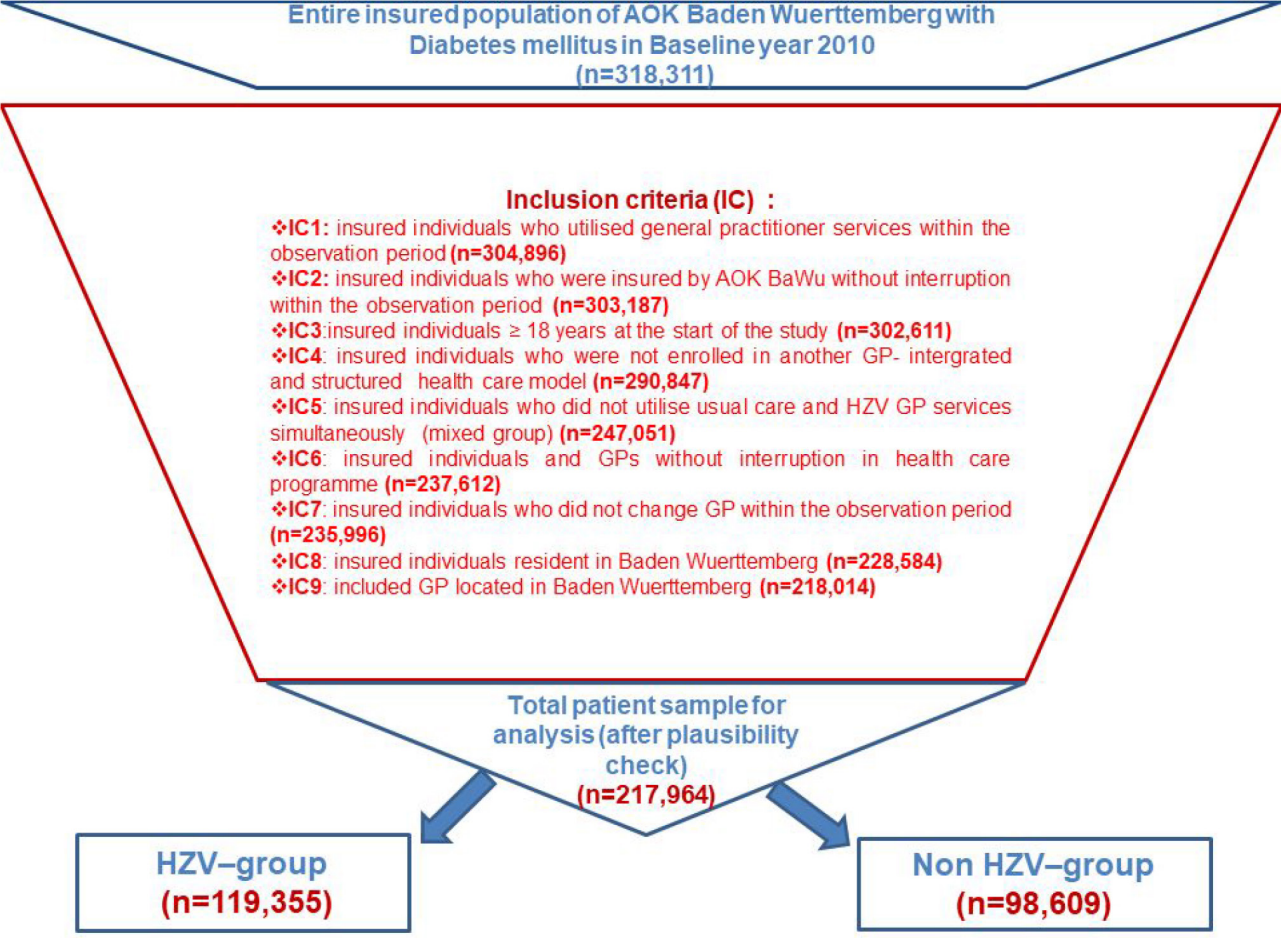
Flow diagram of participation inclusion. AOK = Allgemeine Ortskrankenkasse. HZV = Hausarztzentrierte Versorgung.

In total, 217 964 patients were included in this analysis (Figure 1): 119 355 were enrolled in the GP-centred healthcare programme and 98 609 patients received usual GP care. All baseline characteristics from 2010 are shown in Table 3. Mean age, sex distribution, and comorbidities were similar in both groups. However, there were several baseline differences between patients in the HZV and non-HZV groups. More patients in the usual care group required long-term care (care level >0). A larger percentage (77.3%) of patients in the HZV group participated in the diabetes mellitus DMP compared with patients in the non-HZV group (53.4%). Patients in the HZV group had higher CCI scores and had higher scores on the aDCSI. Baseline practice characteristics on the patient level are also shown in Table 3. GP practices in the HZV group were larger in number of AOK-BaWü patients, were more frequently located in rural areas, and were more often group practices.

**Table 3.** Patient and GP characteristics at baseline (2010)

Patient characteristics	non-HZV, *n* = 98 609, *n* (%)^a^	HZV, *n* = 119 355, *n* (%)^a^	All*, N* = 217 964, *n* (%)^a^
Sex, female	53 668 (54.4)	64 040 (53.7)	117 708 (54.0)
Age, years, mean±SD	69.8±12.0	69.3±11.5	69.5±11.7
DM DMP participation	52 644 (53.4)	92 221 (77.3)	144 865 (66.5)
Care level >0	13 933 (14.1)	13 034 (10.9)	26 967 (12.4)
Charlson Comorbidity Index score, mean±SD	3.2±2.3	3.3±2.3	3.3±2.3
aDCSI score, mean±SD	1.94±1.90	2.01±1.90	1.98±1.90
Arterial hypertension	80 115 (81.2)	97 073 (81.3)	177 188 (81.3)
Ischaemic heart disease	26 892 (27.3)	33 421 (28.0)	60 313 (27.7)
Hospitalisation	30 176 (30.6)	34 480 (28.9)	64 656 (29.7)
**Practice characteristics**			
Size, mean±SD	363.9±194.4	530.6±277.0	455.2±256.9
Group	36 909 (37.4)	57 853 (48.5)	94 762 (43.5)
Urban setting	49 339 (50.0)	56 689 (47.5)	106 028 (48.6)

^a^Unless otherwise stated. aDCSI = adapted Diabetes Complications Severity Index. DM = diabetes mellitus. DMP = disease management programme. SD = standard deviation.

### Primary endpoints

**Figure 2.**
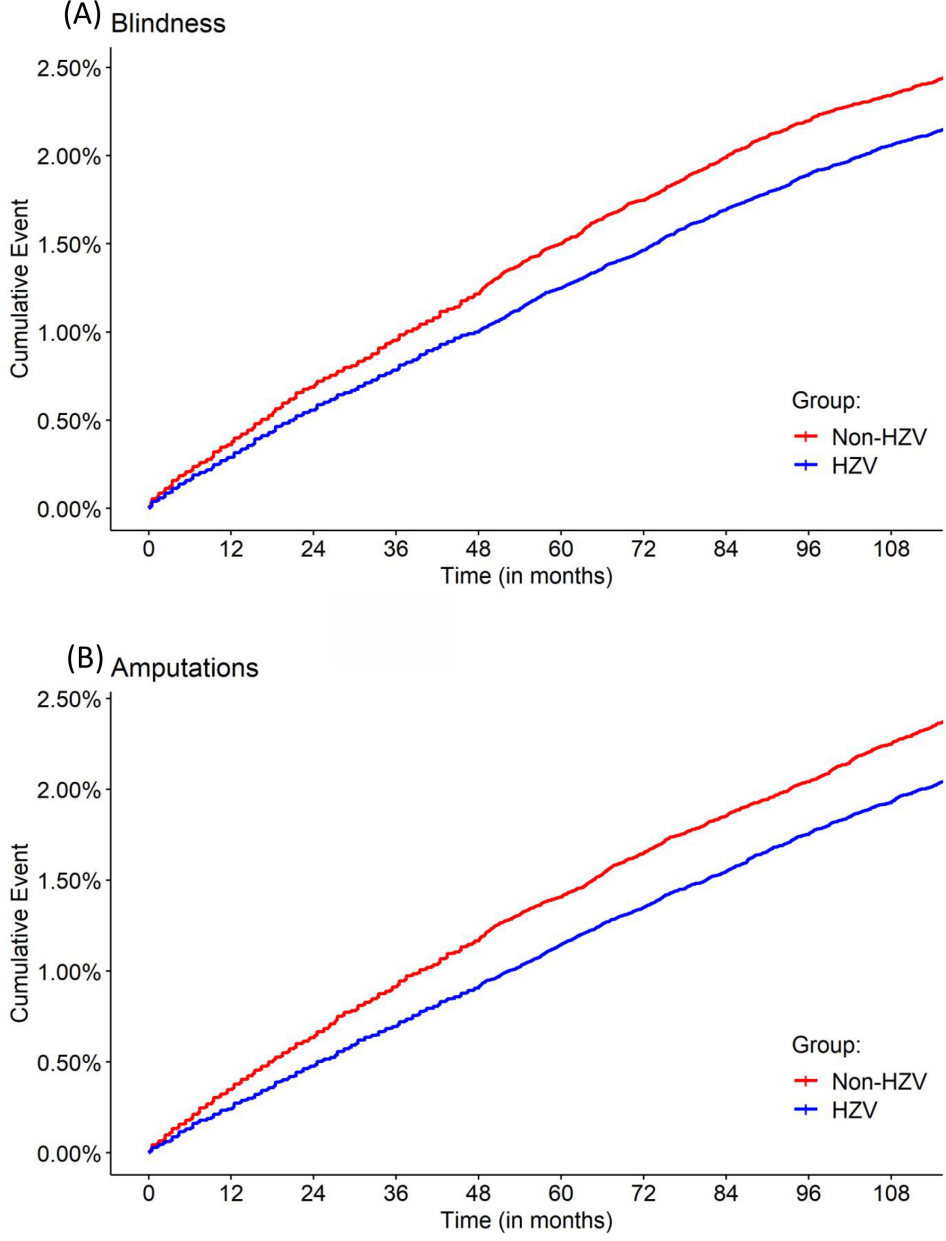
Development of diabetes complications during the 10-year period. **A**) Development of blindness; and **B**) development of amputations. HZV = Hausarztzentrierte Versorgung.

All crude rates of primary endpoints (incident) are shown in Table 4. Cumulative-event curves for blindness and amputation in both groups are presented in Figure 2. The Cumulative-event curves for the remaining endpoints are presented in Supplementary Figures S1–S6.

**Table 4.** Unadjusted rates and hazard ratios for the variable HZV participation from the multivariable Cox models for primary endpoints. Observational period 2011–2020.

Primary endpoints	Non-HZV, unadjusted or crude, *n* = 98 609, %	HZV, unadjusted or crude, *n* = 119 355, %	Hazard ratio (95% CI, *P* value)	Relative risk reduction
Dialysis	3.6	3.7	0.899 (0.880 to 0.918, <0.0001)	**–10.1%**
Blindness	2.5	2.2	0.882 (0.858 to 0.906, <0.0001)	**–11.8%**
Amputation	2.4	2.1	0.797 (0.775 to 0.819*,* <0.0001)	**–20.3%**
Myocardial infarction	14.7	13.7	0.851 (0.842 to 0.860, <0.0001)	**–14.9%**
Stroke	19.0	18.1	0.901 (0.893 to 0.910, <0.0001)	**–9.9%**
Cardiovascular disease	35.1	35.4	0.906 (0.898 to 0.913*,* <0.0001)	**–9.4%**
Hypoglycaemia	18.0	21.7	1.012 (1.002 to 1.022, 0.0186)	**+1.2%**
Mortality	43.7	40.7	0.971 (0.965 to 0.977, <0.0001)	**–2.9%**

Hazard ratios for all covariates and endpoints are shown in Supplementary Tables S1–S8. HZV = Hausarztzentrierte Versorgung.

In the Cox regression models (adjusted for all influence factors and co-variables given in our data: Table 2) participation in the HZV group was significantly protective for all endpoints over the 10-year observation period, with the exception of hypoglycaemia (Table 4). In the HZV group, a 20.3% risk reduction of amputation was demonstrated during the 10-year period, compared with the usual care group. The risk of myocardial infarction was 14.9% lower in the HZV group than in the usual care group, and the risk of dialysis 10.1% lower. In the previously published analysis for the observation period 2011–2014,^2^ patients in the HZV group had an increased risk (risk difference +19%) of hypoglycaemia. In the current study, the risk of hypoglycaemia over the observational period of 10 years had decreased to 'only' 1.2% higher in the HZV group in comparison with usual care. The mortality risk in the HZV group was 2.9% lower than in the usual care. In contrast to the previous analysis from 2011–2014,^2^ the protective effect of HZV enrolment on mortality risk is significant.

Over the 10-year observation period, it can be observed that female sex was significantly protective with regard to all endpoints (see Supplementary Tables S1–S8). Age by decade also had major influence over the development of complications: for most endpoints, age had a significantly negative predictive value, with the exception of dialysis, amputation, and hypoglycaemia, where age had a protective effect (see Supplemetary Tables S1, S3, and S7). Diabetes DMP participation remains a protective prognostic factor, except in the case of amputation, cardiovascular disease, myocardial infarction, and hypoglycaemia (see Supplemetary Tables S1, S2, S5, and S8). The specific diabetes morbidity index, aDCSI, has been shown to have more prognostic value (negative predicting value) regarding the development of complications than the general morbidity index CCI (see Supplementary Tables S1–S8).^11^

## Discussion

### Summary

To the authors’ knowledge, this is the first cohort study on the development of serious clinical endpoints associated with diabetes mellitus over a 10-year period in a structured and coordinated healthcare system in Germany. The results of this study over a 4-year observation period were outlined in the authors’ previous publication.^2^

Over the entire 10-year observation period, the hazard risk to develop studied clinical endpoints was significantly lower in the HZV group, with the exception of hypoglycaemia. The increased risk of hypoglycaemia in the HZV group can be attributed to more intensive diabetes treatment and age of patients. Older adults with type 2 diabetes mellitus are at increased risk of developing hypoglycaemia.^12^ The American Diabetes Association has recently added three new recommendations regarding hypoglycaemia in older people, highlighting individualised pharmacotherapy with glucose-lowering agents that have low risk of hypoglycaemia and proven cardiovascular safety, avoidance of overtreatment, and simplifying treatment regimens while maintaining HbA1c targets.^12^ The risk of hypoglycaemia in the HZV group over the observational period of 10 years decreased compared with the previous publication with 4-years follow-up.^2^ This can be explained by the uptake and implementation of new recommendations in quality circles relating to pharmacotherapy in the HZV.

Participation in diabetes mellitus DMP remained an important independent protective predictor for developing diabetes mellitus-associated endpoints. The impact of DMP over the 10-year period varies depending on the endpoint examined. Compared with the previous 4-year observation period, DMP was a positive predictor for better outcomes^2^ for all endpoints. Currently, DMP participation had a negative impact for amputation, cardiovascular disease, myocardial infarction, and hypoglycaemia. The increased risk of development of cardiovascular disease, myocardial infarction, amputation, and hypoglycaemia for patients who are enrolled in the DMP (diabetes mellitus) is likely owing to the older age of participants and longer diabetes duration in this group.^13^

In recent decades in Germany, there has been a general trend towards a lower incidence of diabetes complications such as amputation, renal failure, or blindness.^13–15^ However, an increased prevalence of complications in older patients with longer diabetes duration can be observed.^13^ The average age in this study population with at least a 10-year diabetes duration (from the beginning of the study) is 69 years. Nonetheless, a decreased risk of developing diabetes-related complications (except hypoglycaemia) was demonstrated in the HZV group. High-quality and structured primary care with close specialist collaboration and a trusting relationship between patient and GP seems to indicate lower risk of developing diabetes-related complications, even in the case of longer diabetes duration.

### Strengths and limitations

This study benefits from the large size of the population-based sample and the inclusion of patients with type 1 and type 2 diabetes. Our data includes inpatient, outpatient, and prescribing data, with no missing values (claims data). The used statistical analysis is very solid, conformed, and appropriate to modelling time-to-event analysis.

This study had several potential limitations. First, the claims dataset did not contain clinical data or the duration of a patient’s diabetes diagnosis before 2010. Second, although data on drug therapies would have been available, no generally accepted graduation of drug therapies exists for use within the context of our analysis.

It should be noted that participation in HZV was not randomised, but voluntary. As such, there is a natural selection bias, which was minimised by adjustment for potential influence factors (Table 3) given in the claims data.

### Comparison with existing literature

The results of the present study are similar to international published studies regarding the development of diabetes-related complications and the effects of diabetes management in the primary care context.

Several studies have demonstrated that patients with diabetes benefit from participating in an integrated health delivery system.^16–19^ International studies have shown that participants with diabetes, who were enrolled in an integrated healthcare programme, were less likely to be readmitted within 90 days of discharge.^17^ Integrated health care was effective in improving clinical outcomes, reducing the general outpatient clinic utilisation rate over a 12-month period, and lowering the mortality rate.^19^

Dusheiko *et al* came to the conclusion that GP practices with a higher quality of diabetes care had fewer emergency admissions for short-term complications of diabetes.^20^ Over time, after controlling for national trends in admissions, improvements in the quality of care delivery in general practice were associated with a reduction in admissions.^21^ Roth *et al* showed that low rates of hypoglycaemia, lower limb amputations, and good glycaemic control in secondary care patients indicate a good structure of patient care.^13^ Furthermore, O’Conner *et al* demonstrated in their observational study the effect of pay-for-performance on diabetes mellitus management, resulting in improved rates of recording of clinical and biochemical parameters.^6^

The authors could not find a comparable study on the time-dependent development of diabetic complications.

### Implications for research and practice

The treatment goals for diabetes are to prevent or delay complications and optimise quality of life.

In Germany, as well as worldwide, a large portion of care for the chronically ill is provided by GPs who often strive to enhance chronic care. The findings of this study, that the strengthening of structured primary care is associated with a delay or reduction in diabetes complications, may be of interest for future service planning and delivery.

This study of the development of diabetes complications over a 10-year observation period within the GP-centred healthcare programme (HZV) demonstrates a highly sustainable healthcare model, in comparison with classic pay-for-performance models within primary care, which have been shown to reach a plateau or have modest and variable effects on the quality of health care provided by GPs.^22,23^

The GP-centred healthcare programme model could be introduced into other countries with a functioning primary care system but fragmentary and lacking chronic disease management programmes and/or fragmentary primary and secondary care.

## Data

According to the current project contract for carrying out the HZV (GP-centred care) evaluation, data may only be used for the purpose of evaluating the HZV. Access and user control ensure that access to data from other clients is not possible. Use of data for purposes other than those described in the contract is not permissible.
